# Pre-microRNA and Mature microRNA in Human Mitochondria

**DOI:** 10.1371/journal.pone.0020220

**Published:** 2011-05-26

**Authors:** Eric Barrey, Gaelle Saint-Auret, Blandine Bonnamy, Dominique Damas, Orane Boyer, Xavier Gidrol

**Affiliations:** 1 Unité de Biologie Intégrative des Adaptations à l'Exercice – INSERM U902, Genopole Evry, France; 2 Biopuces et Génomique Fonctionnelle (Biomics), Direction des Sciences du Vivant, CEA, Grenoble, France; University of Colorado, Boulder, United States of America

## Abstract

**Background:**

Because of the central functions of the mitochondria in providing metabolic energy and initiating apoptosis on one hand and the role that microRNA (miRNA) play in gene expression, we hypothesized that some miRNA could be present in the mitochondria for post-transcriptomic regulation by RNA interference. We intend to identify miRNA localized in the mitochondria isolated from human skeletal primary muscular cells.

**Methodology/Principal Findings:**

To investigate the potential origin of mitochondrial miRNA, we in-silico searched for microRNA candidates in the mtDNA. Twenty five human pre-miRNA and 33 miRNA aligments (E-value<0.1) were found in the reference mitochondrial sequence and some of the best candidates were chosen for a co-localization test. *In situ* hybridization of pre-mir-302a, pre-let-7b and mir-365, using specific labelled locked nucleic acids and confocal microscopy, demonstrated that these miRNA were localized in mitochondria of human myoblasts. Total RNA was extracted from enriched mitochondria isolated by an immunomagnetic method from a culture of human myotubes. The detection of 742 human miRNA (miRBase) were monitored by RT-qPCR at three increasing mtRNA inputs. Forty six miRNA were significantly expressed (2^nd^ derivative method Cp>35) for the smallest RNA input concentration and 204 miRNA for the maximum RNA input concentration. In silico analysis predicted 80 putative miRNA target sites in the mitochondrial genome (E-value<0.05).

**Conclusions/Significance:**

The present study experimentally demonstrated for the first time the presence of pre-miRNA and miRNA in the human mitochondria isolated from skeletal muscular cells. A set of miRNA were significantly detected in mitochondria fraction. The origin of these pre-miRNA and miRNA should be further investigate to determine if they are imported from the cytosol and/or if they are partially processed in the mitochondria.

## Introduction

The mitochondria house the vital energetic function to provide ATP in most of the cells and especially in muscle by oxidative phosphorylation. Another important role is to initiate intrinsic apoptosis pathway. The mitochondria are also involved in cytosolic calcium regulation [1] and in synthesis of the heme and steroid hormones. All these functions require several pathways and numerous enzymatic and regulation processes. Despite the fact that the mitochondrion has its own genomic, transcriptomic and proteomic materials, it is not entirely autonomous to produce all the proteins required for its full functionality [2]. There are only 13 mitochondrial genes coding for sub-units of the respiratory chain complexes in the mitochondrial genome. However, more than one thousand proteins have been identified in the mammalian mitochondria [3]. All of them have to be imported through the outer mitochondrial membrane by the translocase outer mitochondrial (TOM) complex and through the inner membrane by one of the following processes: translocase of the inner membrane (TIM22, TIM23) or oxidative folding pathway of the intermembrane space (OXA) [4]. Mitochondrial protein trafficking is intense and finely regulated. This trafficking is extended to tRNA through a distinct molecular mechanism. The mitochondrial genome codes for 22 tRNA and most of them can be mutated in human mitochondrial diseases [5]. One of these, MERRF (Myoclonic Epilepsy and Ragged Red Muscle Fibers), is due to a tRNA_Lys_ mutation. The cytosolic tRNA importation in the mitochondria was studied *in vitro* and *in vivo* models to cope with this mitochondrial tRNA_Lys_ deficiency. *In vitro* models demonstrated the capacity of the mitochondria to import yeast or human tRNA_Lys_ dependent on amino-acylation and the presence of the precursor lysyl-tRNA synthase in the mitochondria. Other protein factor(s) could promote the importation of the 5S rRNA [6]. It was also demonstrated that the cytosolic tRNA_Gln_ was imported into mitochondria using ATP but did not require any cytosolic factor or protein translocation system [7]. Again these important results showed the capacity of the mitochondria to import macromolecules such as ribosomic and transfer RNA by different translocation systems.

In order to regulate this intense trafficking between the cytosol and mitochondria, and to synchronize nuclear and mitochondrial functions, several levels of regulation are operating in the mitochondria. The following transcription factors are involved in the communication from the nucleus to the mitochondria: TFAM, TFB1M, TFB2M, p53, NF-kB, AP1, CREB [8]. In addition, nuclear receptors of oestrogen, glucocorticoid and thyroid hormones can be translocated into mitochondria to regulate mitochondrial gene transcription. The retrograde communication from the mitochondria to the nucleus seems to occur in case of mitochondrial dysfunction and in some pathologies such as cancer [9]. In skeletal mammalian myoblasts and in human pulmonary carcinoma cells, mitochondrial retrograde signaling seems to occur through cytosolic [Ca2+] changes [10], [11]. The retrograde signaling could be realized by the translocation of some mitochondrial proteins to the cytosol like in apoptosis where cytochrome c is the signal sent for activating the caspases cascade. In case of mitochondrial dysfunction, the retrograde signaling pathway is activated through the nuclear transcriptomic factors cascade RTG2p and RTG1/3p [12]. Post-transcriptional regulations occurs in mitochondria as well. For instance proteins binding to polyadenylated mRNA have been reported [13]. In the rat brain development, factors were able to bind mRNA of COXIII and COXIV in mitochondria [9], [13].

Post-transcriptomic regulation by the microRNA (miRNA) was discovered in *C. elegan*s (small RNA lin-4) [14] and then in *Arabidopsis* [15]. Multiple eukaryotes including fungi, plants, protozoans and metazoans produce RNA silencing systems that are involved in many gene regulation processes during proliferation, differentiation and pathologies like cancers [16], [17]. According to the literature, miRNA has never been observed in human mitochondria. This cell organelle plays a vital cellular function which requires a fine tuning of post-transcription regulation as any other pathways. We hypothesized that some miRNA could be imported and/or processed in the mitochondria for post-transcriptomic regulation of mitochondrial and may be nuclear genes by RNA interference. These putative mitochondrial miRNA could actively contribute to the cross-talk with the nucleus. Recently, microRNA were described in rat and mouse liver isolated mitochondria [18], [19], but without demonstrating any co-localization of miRNA in the mitochodria.

The aims of the study was to co-localize miRNA candidates in the human mitochondria. After predicting miRNA candidates by *in silico* analysis, we co-localized the best pre-miRNA and miRNA candidates in the human mitochondria of skeletal muscular cells by using *in situ* hybridization. Then, we investigate the expression of the human miRNA panel in highly purified mitochondria fraction. Finally, we looked for potential miRNA targets in the mitochondrial genome by *in silico* analysis.

## Results

### Pre-miRNA and miRNA candidates in the mitochondrial genome

**Figure 1:**
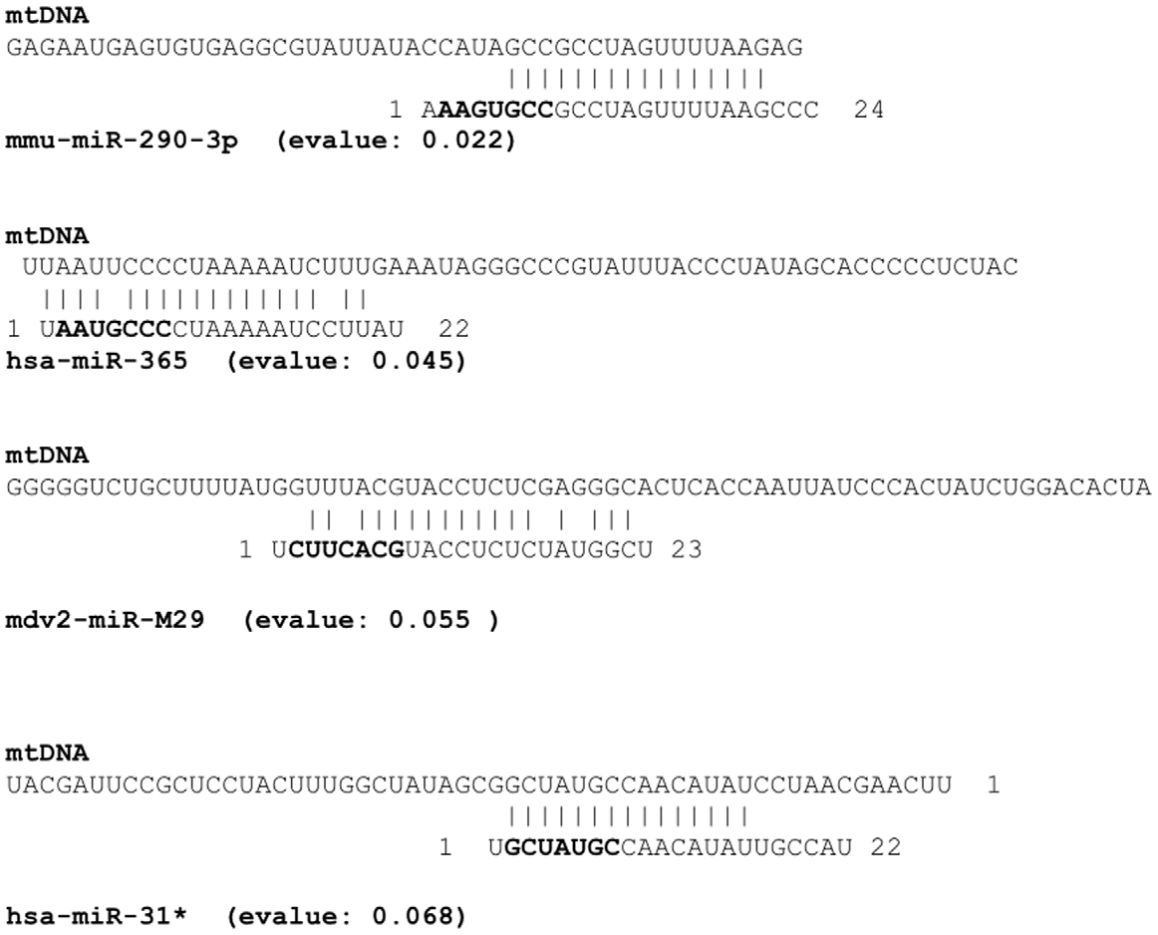
Examples of alignment results of human mature miRNA 365 and 31. The nucleotides in bold letters indicate the seed region of the miRNA.

**Figure 2:**
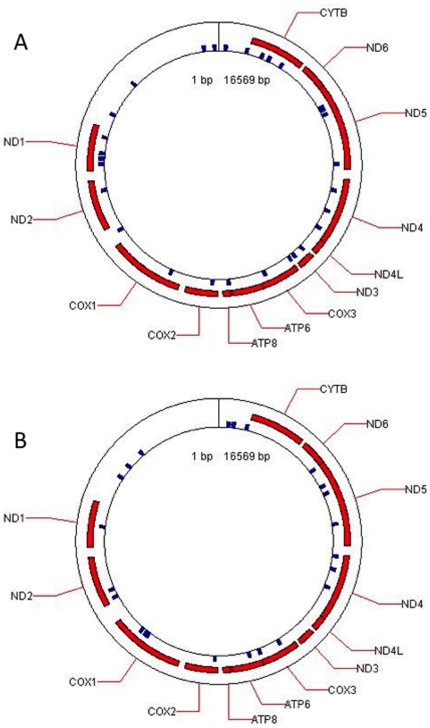
Mitochondrial map of the human pre-mature (A) and mature miRNA (B) candidates identified by alignements between the reference mitochondrial sequence and miRBase data. (A) The locations of the human pre-miRNA described in Table 3 are represented as blue squares on the mitochondrial genome. (B) The locations of the human mature miRNA described in Table 4 are represented as blue squares on the mitochondrial genome.

To evaluate the possibility of miRNA coding sequences in the mitochondrial genome, we searched the mitochondrial reference sequence using miRBase search tool. We obtained a list of 33 human pre-miRNA (Table 1) and 25 miRNA (Table 2). The most significant alignments with human miRNA were obtained with four pre-miRNA (pre-mir-302a, pre-let-7b, pre-mir-1267 and pre-mir-1296; E-value <0.1) and with the two miRNA (mir-365 and mir-31; E-value <0.1) Examples of alignment results show that for some candidates the seed region was perfectly aligned and some others had one or two nucleotide differences (Figure 1). These pre-miRNA and miRNA candidates were mapped on the mitochondrial genome (Figure 2A and 2B). The presence of miRNA and two pre-miRNA, mir-365, let-7b and pre-mir-302a and pre-let7b, in myoblastic mitochondria was further evaluated by in situ hybridization.

**Table 1:** List of human pre-miRNA aligned with the human mtDNA reference sequence at E-value <1.

miRNA ID	Accession #	Strand	Score	Evalue
hsa-mir-1267	MI0006404	−	145.6	0.022
**hsa-mir-302a**	MI0000738	+	143	0.035
**hsa-let-7b**	MI0000063	−	138	0.055
hsa-mir-1296	MI0003780	+	135.9	0.065
hsa-mir-522	MI0003177	−	131.1	0.13
hsa-mir-7-2	MI0000264	+	128.5	0.14
hsa-mir-632	MI0003647	+	126.7	0.21
hsa-mir-548k	MI0006354	+	122.2	0.3
hsa-mir-541	MI0005539	+	124.4	0.31
hsa-mir-1256	MI0006390	+	121.4	0.32
hsa-mir-576	MI0003583	−	121.6	0.39
hsa-mir-412	MI0002464	+	121.8	0.4
hsa-mir-1273	MI0006409	−	119.7	0.46
hsa-mir-320a	MI0000542	−	121.4	0.47
hsa-mir-595	MI0003607	−	120.1	0.48
hsa-mir-1275	MI0006415	−	120.8	0.52
hsa-mir-526b	MI0003150	+	119.3	0.61
hsa-mir-320b-1	MI0003776	+	118.6	0.69
hsa-mir-1183	MI0006276	+	117.7	0.7
hsa-mir-1243	MI0006373	−	117	0.73
hsa-mir-548d-2	MI0003671	+	116.3	0.77
hsa-mir-1322	MI0006653	+	118.6	0.78
hsa-mir-329-1	MI0001725	+	117.4	0.8
hsa-mir-329-2	MI0001726	+	117	0.81
hsa-mir-548f-5	MI0006378	−	116.8	0.81
hsa-mir-518f	MI0003154	−	115.9	0.89
hsa-mir-1286	MI0006348	+	116.8	0.9
hsa-mir-365-2	MI0000769	+	114	0.9
hsa-mir-579	MI0003586	−	114.8	0.91
hsa-mir-26a-2	MI0000750	+	116	0.92
hsa-mir-548a-2	MI0003598	−	114.5	0.96
hsa-mir-532	MI0003205	−	114.6	1
hsa-mir-889	MI0005540	−	115.7	1

The miRNA candidates used for in situ hybridization are indicated in bold.

**Table 2:** List of human mature miRNA aligned with the human mtDNA reference sequence at E-value <1.

miRNA ID	Accession #	Strand	Score	Evalue
**hsa-miR-365**	MI0000767	+	137	0.045
hsa-miR-31*	MI0000089	−	133.8	0.068
hsa-miR-652	MI0003667	+	129.6	0.12
hsa-miR-557	MI0003563	+	125.4	0.19
hsa-miR-590-5p	MI0003602	+	124.3	0.23
hsa-miR-7-2*	MI0000264	+	123	0.27
hsa-miR-516b	MI0003167	+	122.4	0.29
hsa-miR-765	MI0005116	−	121.4	0.35
hsa-miR-127-5p	MI0000472	+	121.1	0.35
hsa-miR-190b	MI0005545	−	121.1	0.37
hsa-miR-637	MI0003652	+	120.6	0.34
hsa-miR-936	MI0005758	+	118.1	0.51
hsa-miR-582-3p	MI0003589	−	117.7	0.54
hsa-miR-451	MI0001729	+	117.5	0.55
hsa-miR-606	MI0003619	+	117.5	0.58
hsa-miR-198	MI0000240	−	117	0.59
hsa-miR-328	MI0000804	+	115.5	0.72
hsa-miR-132*	MI0000449	+	115	0.76
hsa-miR-186	MI0000483	−	114	0.86
hsa-miR-10b*	MI0000267	+	113.7	0.9
hsa-miR-197	MI0000239	+	112.9	0.99
hsa-miR-589*	MI0003599	−	112.9	0.91
hsa-miR-556-3p	MI0003562	−	112.8	1
hsa-miR-135a	MI0000452	−	112.4	1
hsa-miR-582-5p	MI0003589	+	112.4	1

The miRNA candidate used for in situ hybridization is indicated in bold. The miRNA labelled with a * were the minor mir* sequence corresponding to the complementary sequence of the mature miRNA of the same number.

### *In situ* hybridization demonstrated the presence of pre-miRNA and miRNA in mitochondria

**Figure 3:**
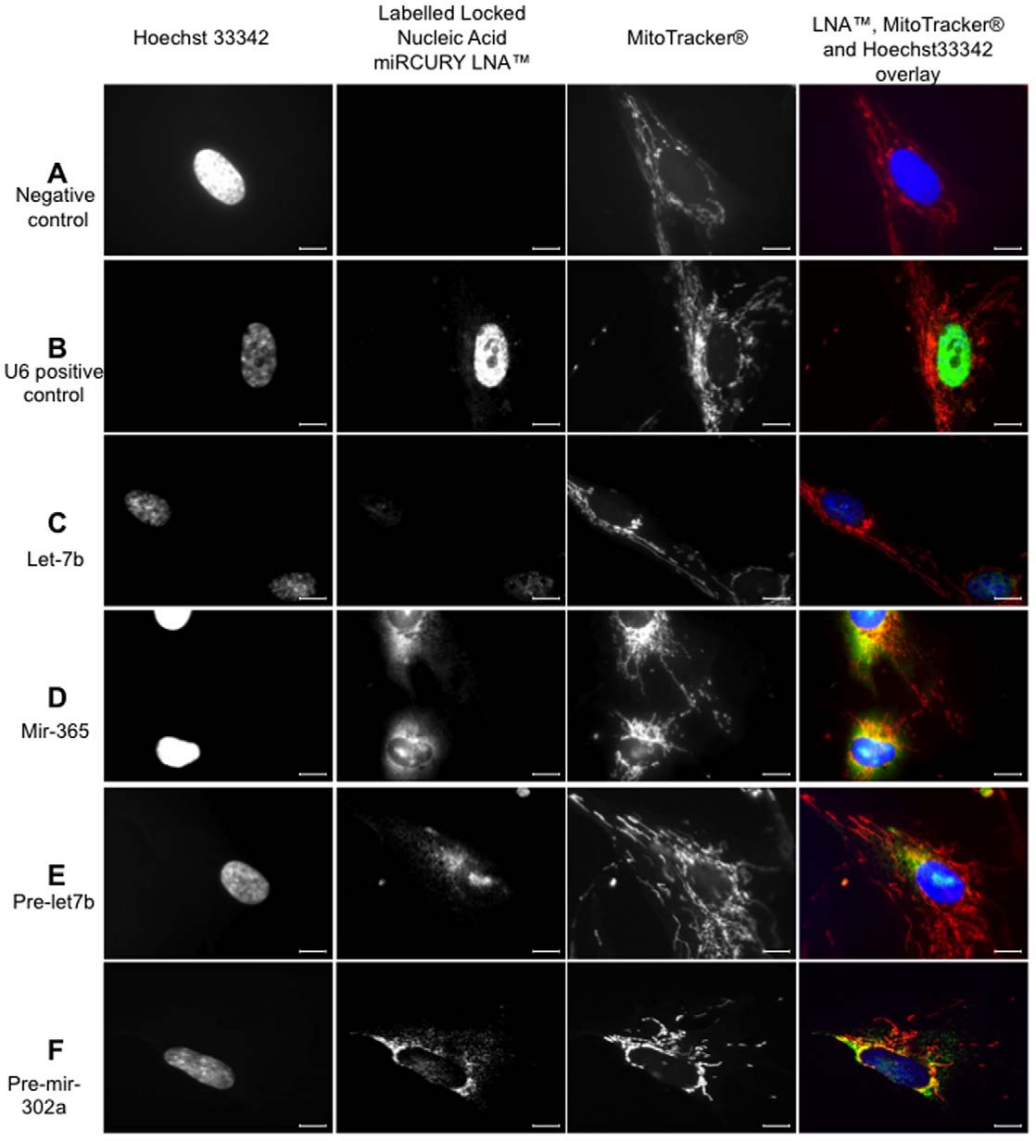
*In situ* hybridization pattern of digoxigenin-labeled Locked Nucleic Acid (LNA) for specific miR and pre-miR in human skeletal muscle myoblasts cells observed in classic optic microscopy. Using locked nucleic acid (LNA) probes digoxigenin labelled, we determined the in situ hybridization pattern of mir and pre-mir. Hoechst 33342 staining of nuclei (lane 1), specific signal of scramble miRNA (A; negative control), U6 small nuclear RNA (B; positive control), let-7b (C), mir-365 (D), pre-let-7b (E) and pre-mir-302a (F) probes (lane 2) and MitoTracker® Red CM-H_2_XRos staining of respiring mitochondria (lane 3) are represented in gray scale. All these images were acquired using an Olympus BX61 straight microscope controlled with Metamorph software (Molecular Devices, Downington, PA19335) using a 100× oil-immersion objective. In the overlays (lane 4) provided by Image J software, positive in situ hybridization signals are visualized in green, respiring mitochondria signal in red and nuclei staining in blue. Yellow staining suggests co-localization of LNA probes (green fluorescence) and MitoTracker® Red CM-H_2_Ros (red fluorescence). Scale bars = 10 µm. Images are not scaled to the same intensity range. Positive in situ hybridization signals were normalized by scramble miR signal intensity (negative control).

**Figure 4:**
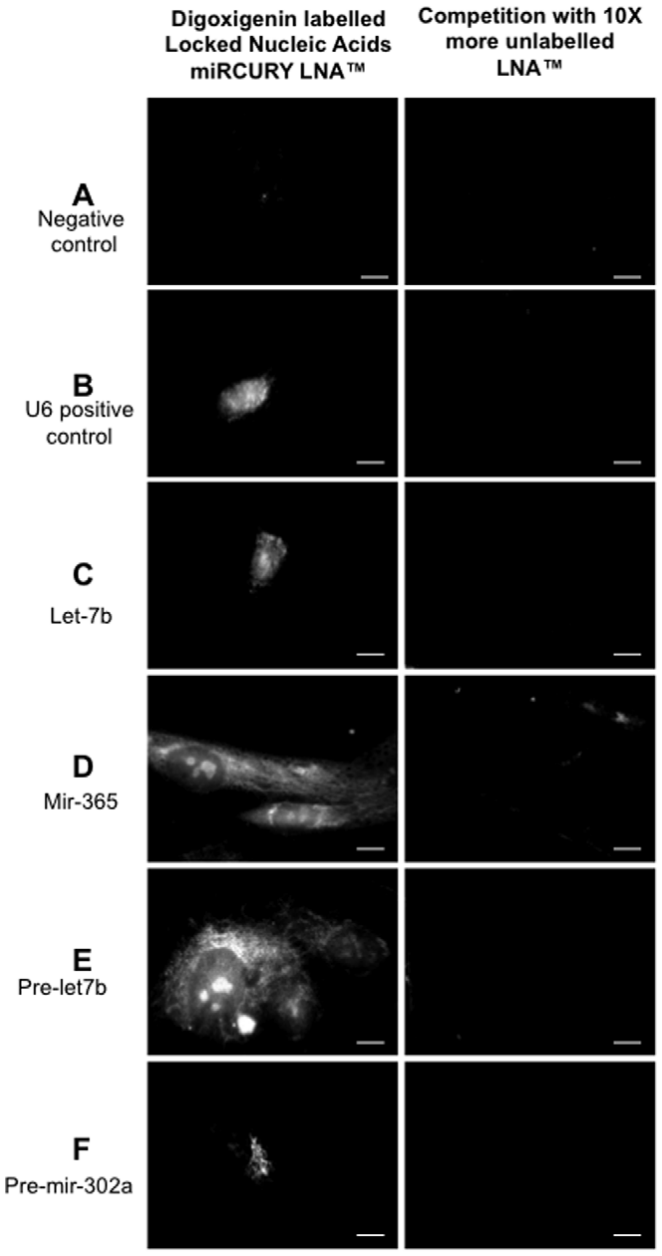
Specificity of the locked nucleic acid (LNA) probes for the detection of miR and pre-miR using in situ hybridization. Using 2.5 pmol of locked nucleic acid (LNA) probes prelabelled with digoxigenin, we determined in situ hybridization patterns of scramble miR (A; negative control), U6 small nuclear RNA (B; positive control), let-7b (C), mir-365 (D), pre-let-7b (E) and pre-mir-302a (F) probes (lane 1). In the second lane, Digoxigenin-labeled probes were competed with excessive amount (25 pmol ie 10× more) of unlabeled probes. Most of the signal observed in the panels of the first lane are absent from the panels in the second lane. Scale bars = 10 µm. Images are not scaled to the same intensity range. Positive in situ hybridization signals showed were normalized by scramble mirna signal intensity (negative control).

**Figure 5:**
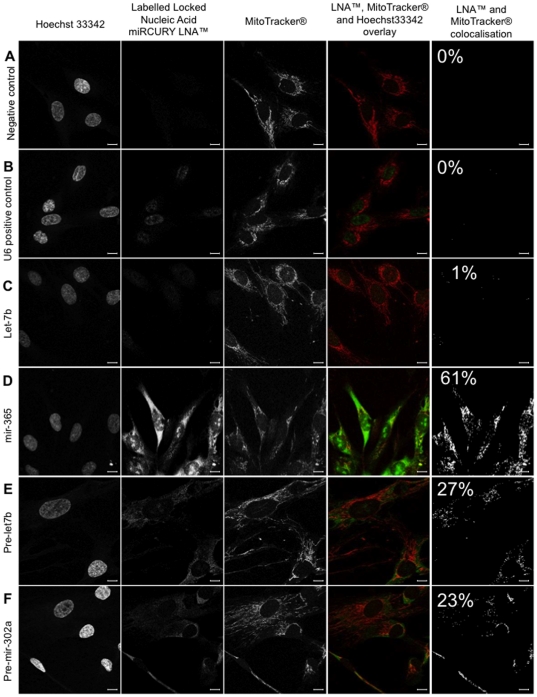
In situ signal of mir-365, pre-mir-let7b and pre-mir-302a co-localized with functioning mitochondria in human myoblasts observed in confocal microscopy. Using locked nucleic acid (LNA) probes, we performed in situ hybridization to localized mir-365, mir-let-7b, pre-let-7b and pre-mir-302a within the cell. Hoechst 33342 staining of nuclei (lane 1), specific signal of scramble miR (A; negative control), U6 small nuclear RNA (B; positive control), let-7b (C), mir-365 (D), pre-let-7b (E) and pre-mir-302a (F) provided by locked nucleic acid (LNA) probes (lane 2) and MitoTracker® Red CM-H_2_XRos staining of functioning mitochondria (lane 3) are represented in gray scale. All these images were acquired with a Leica TSC-P2 confocal microscope using a 63× oil-immersion objective. A sequential mode for three colour of acquisition (FITC for LNA probes signals, Dapi for Hoechst staining and Cy3 for MitoTracker® Red CM-H_2_XRos) has been used. In the overlays (lane 4) provided by Image J software, positive in situ hybridization signal are visualized in green and respiring mitochondria signal in red. Yellow corresponded to red and green overlay. The pixels with co-localized signals (lane 5) from functioning mitochondria and specific LNA probe for mir or pre-mir are determined using the Image J MBF plugging “co-localization highlighter”. Scale bars = 10 µm. The raw images are showed in the figure. The percentage of pixels with co-localized miRNA and mitochondrial signals were determined using Isodata plugging threshold (Image J software) and indicated on the figure (% in lane 5).

To demonstrate that these miRNA were localized in mitochondria, we performed *in situ* hybridization using specific labelled locked nucleic acid sequences that specifically hybridized with corresponding miRNA. The mitochondria were labelled with a selective fluorescent probe which passively diffused across the plasma membrane and accumulate in active mitochondria (Red MitoTracker®). Thus, the staining of the mitochondria by the MitoTracker® was a proof of their integrity and functionality. The images were then analyzed by fluorescent conventional microscopy (Figure 3) and then confirmed by confocal microscopy (see below). As positive control we used a LNA targeting the small nuclear RNA, RNU6B, (Figure 3A). A LNA targeting scramble miRNA, was used as negative control and did not generate any signal (Figure 3B). Mir-365, pre-mir-302a and pre-let7b specific fluorescent LNA were clearly co-localized in perinuclear mitochondria, as demonstrated by the yellow signal one could observed in that area (Figure 3D, E, F). Mir 365 exhibited a strong signal in the mitochondria but also in some areas of the nucleus (Figure 3D). The same observation was made for pre-let-7b (Figure 3E). Surprisingly, let-7b localized only in some points of the nucleus (Figure 3C). To further evaluate the specificity of the labels, we chased fluorescent LNA with a 10× excess of unlabelled LNA. As demonstrated in Figure 4, *in situ* hybridization was no longer observed in presence of unlabelled LNA, thus demonstrating the specificity of the *in situ* hybridization. Although specific, the co-localization of miRNA and mitochondria that we observed could be biased by the conventional microscopy. As a final demonstration of miRNA co-localization in the mitochondria, we analyzed LNA-mediated specific miRNA *in situ* hybridization by fluorescent confocal microscopy (Figure 5). We confirmed the intense co-localization of mir-365 in the mitochondria and some local areas in the nucleus (Figure 5 D). Strikingly, we also observed that two pre-miRNAs, pre-mir302a and pre-let-7b were located within mitochondria surrounding the nucleus (Figures 5E, F). As in coventional microscopy, some traces of pre-let-7b were observed in the nucleus (Figure 5E). On the opposite, the mature form of let-7b was poorly detected in the mitochondria (co-localization in 1% of the pixels) but mainly in the nucleus (Figure 5C). Since *in situ* hybridization experiments clearly established the presence of at least one miRNA and two pre-miRNA in human mitochondria, we next analyzed the small RNA content in highly purified mitochondria isolated from human myotubes.

### RNA content in isolated mitochondria

Myotubes were obtained through *in vitro* differentiation of primary human myoblasts. Mitochondria were isolated using supramagnetic microbeads conjugated to the monoclonal antibody against the translocase protein of the mitochondrial outer membrane TOM22 (Magnetic antibody cell sorting method = MACS method). The enrichment in mitochondria was then evaluated in this fraction.

The comparison of the equal amount of proteins in the isolated mitochondria and the total cell extract showed the high enrichment in mitochondrial proteins, 22-kDa translocase outer mitochondrial membrane (TOM22) and ATP synthase. While two cytosolic proteins, GAPDH and ACTB were clearly depleted in the mitochondrial fraction (Figure S1). In addition, the high values of mitochondrial to nuclear DNA ratios obtained for ND4 and CYTB genes confirmed this high mitochondria enrichment (Table S1). Furthermore, the relative expression of the two mitochondrial genes ND4 and CYTB were respectively 724 to 540 times greater in the mtRNA extract than in the cytosolic RNA extract (Table S2). This result showed the high enrichment of mtRNA in the mitochondrial fraction. The relative expression of the two nuclear genes GAPDH and HIST2H2AA3 were respectively 1.5 10^−3^ to 5.83 10^−5^ lower in the mtRNA extract than in the cytosolic extract (Table S2). This indicated a very low contamination of the mitochondrial fraction by the genomic mRNA.

The integrity of mitochondrial membranes and ultrastructure seemed to be preserved as well, as demonstrated on the transmission electron microscopy images (Figure S2 A; Figure S2 B; Figure S2 C). It was possible to observe a continuous double membranes, cristae, a normal matrix density and the supramagnetic microbeads conjugated to the antibody anti-TOM22 bound to the translocase outer membrane protein. The mitochondria integrity confirmed the low risk of mtRNA contamination by cytosolic miRNA during preparation.

The mitochondrial small RNA concentration was measured and compared to the human miRNA reference panel. There was no significant difference in the small RNA concentration and average small RNA size between the mitochondrial fraction and the miRNA reference panel (Table 3). The miRNA in the mitochondrial fraction represented 29.1% of the total small RNA, a significantly lower (p<0.05) concentration than the miRNA reference panel (Table 3). The microfluidic electrophoresis of mitochondrial small RNA revealed several peaks below 70 nt (Figure S3 A): a peak at 40–45 nt, another peak at 50–55 nt and a peak at 60–65 nt. In comparison with the miRNA reference panel (Figure S3 B), we did not observe a sharp peak at 40 nt but only a small amount of miRNA according to the surface under the curve. This quantitative measurement indicated the presence of a significant amount of small RNA in the mitochondria, including 29% of potential miRNA sequences. We proposed to identify these miRNA by RT-qPCR.

**Table 3:** Concentration of small RNA measured by microfluidic electrophoresis in the mitochondrial RNA extract and comparison with the human miRNA reference panel.

Measures	Mitochondrial RNA extract	Reference panel miRNA	Significance
Small RNA (0-276 nt) concentration [pg/µl]	870.91 (313.76)	1066.12 (299.16)	NS
small RNA avarage size [nt]	62.11 (7.80)	67.88 (7.80)	NS
Percentage of miRNA (10 to 40 nt) (%)	29.1 (6.93)	70.75 (6.32)	p<0.0001
miRNA avarage size [nt]	29.33 (2.22)	23.81 (1.16)	p<0.05

### MiRNA detection in the mitochondria

**Figure 6:**
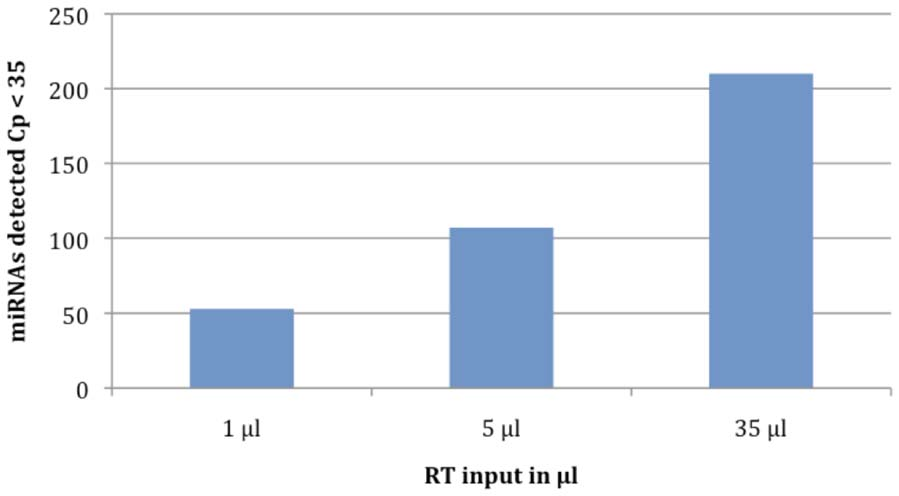
Number of significant miRNA detected in three increasing mitochondrial mtRNA inputs. The number of assays giving a significant signal (Cp<35) was 46, 106 and 204 respectively. The maximum number of assays giving a significant signal represents 27% of the total amount of miRNA assays tested. The number of miRNAs did as expected followed the amount of RNA input and demonstrated the quality of the mitochondrial mRNA extract for miRNA profiling.

The amount of 742 human miRNA (miRBase) were monitored by RT-qPCR analysis of specific miRNA assays and controls. The analyses were conducted on three increasing quantity of the mitochondrial RNA input (Figure S4 and S5). There was no sign of RT-qPCR inhibitors. The mtRNA extracted from purified mitochondria appeared to be a good source for miRNA profiling. Forty six, 54 and 160 miRNA were significantly detected (Cp<35; 2^nd^ derivative method) for the three increasing mtRNA inputs, respectively (Figure 6). The list of all the significant miRNA detected in the mitochondrial mRNA extract was given Table 4 and Table S3. The top-20 most detected microRNA in the mitochondria of the human myotubes were mir-720, 133b, 1974, 24, 133a, 125a-5p, 1979, 103, 125b, 103, 221, 23a, let-7b, 423-3p, 106a, 23b, 92a, 193b and 365. Among them, the mir-365, detected by *in situ* hybridization, was confirmed. Let-7b localization was also confirmed and as well as let-7b*, let-7c, let-7d, let-7d*, let-7e, let-7f, let-7g, let-7i and let-7i* were significantly detected. Mir-302a was detected at a non significant level (35>Cp>40). We significantly detected 6 miRNA known for their functions in controlling myoblastic proliferation and differentiation: mir-133a, mir-206, mir-1, mir-195, mir-181a, mir-181b. The miRNA let-7 familly was very represented. Many miRNA with various functions were detected in a robust and reliable fashion in the mitochondrial RNA extract. If so many miRNA were present in the mitochondria, their potential targets in the mitochondrial genome should be investigated.

**Table 4:** List of microRNA significantly detected in the mitochondrial mRNA at three increasing mtRNA inputs (1, 5 and 35 µL).

MicroRNA	1 µl	5 µl	35 µl	Count
hsa-miR-720	25.95	23.17	20.64	3
hsa-miR-133b	28.46	25.80	22.80	3
hsa-miR-1974	28.59	26.74	23.50	3
hsa-miR-24	28.88	26.66	23.75	3
hsa-miR-133a	30.05	27.65	24.43	3
hsa-miR-125a-5p	31.56	28.70	25.60	3
hsa-miR-1979	31.31	28.65	25.60	3
hsa-miR-103	31.14	28.79	25.90	3
hsa-miR-125b	31.87	28.90	25.91	3
hsa-miR-103	31.34	28.79	26.24	3
hsa-miR-221	31.57	29.29	26.25	3
hsa-miR-23a	32.46	29.51	26.29	3
hsa-let-7b	33.52	29.45	26.83	3
hsa-miR-423-3p	31.59	29.84	26.99	3
hsa-miR-106a	32.31	29.69	27.03	3
hsa-miR-23b	32.99	30.10	27.19	3
hsa-miR-92a	32.96	29.93	27.52	3
hsa-miR-193b	32.66	30.63	27.55	3
hsa-miR-365	33.12	30.33	27.55	3
hsa-miR-93	32.21	30.44	27.62	3
hsa-miR-532-3p	33.11	31.44	27.81	3
hsa-miR-20a	32.86	30.91	27.82	3
hsa-miR-149	34.25	30.79	28.08	3
hsa-miR-181a	33.53	30.94	28.19	3
hsa-miR-503	33.14	31.27	28.21	3
hsa-miR-210	33.90	31.50	28.50	3
hsa-miR-107	33.56	31.99	28.52	3
hsa-miR-574-3p	33.18	31.69	28.65	3
hsa-miR-34a	33.37	31.10	28.65	3
hsa-let-7g	34.58	31.51	28.68	3
hsa-miRPlus-D1033	33.70	31.98	28.85	3
hsa-miR-19b	34.71	32.00	28.87	3
hsa-miR-197	33.95	31.91	28.89	3
hsa-miR-324-3p	32.35	31.52	28.96	3
hsa-miR-127-3p	33.85	32.14	28.99	3
hsa-miR-324-5p	34.01	31.98	29.00	3
hsa-miR-484	34.47	31.91	29.17	3
hsa-miR-151-5p	34.93	32.92	29.56	3
hsa-miR-486-5p	34.32	32.49	29.57	3
hsa-miR-542-5p	34.24	32.48	29.60	3
hsa-miR-199a-5p	34.44	32.69	29.97	3
hsa-miR-501-3p	34.00	33.17	30.00	3
hsa-miR-675*	34.94	32.56	30.15	3
hsa-miR-134	33.74	32.99	31.44	3
hsa-miR-490-3p	34.42	33.36	32.84	3
hsa-miR-598	34.93	34.44	34.27	3

Assays must have Cp <35 and distinct melting curves to be included in the list. Any assays that showed multiple peaks have been excluded from the data set. Forty six miRNA were significantly detected in the three increasing mRNA inputs. It represented 6% of the human miRNA set tested.

### Potential miRNA target sites in the mitochondrial genome

*In silico* results identified a total of 169 potential targets of miRNA in the reference mtDNA (E-value<0.1) and 80 were significant at E-value<0.05 (Table S4). The ND6 gene had 38 target sites twice more than the other genes like CYTB, ND1. Other genes like ND4L, ND4 and COX1 had a lower number of target sites (<10). Among these potential target sites, some were targeted by 46 miRNA significantly detected in the mitochondria (Table S4 column ‘tested miRNA’). Let-7b seemed to have several potential mitochondrial gene targets: ATP6, ATP8, COX2 and ND5. Other members of the let-7 familly, detected by RT-qPCR, had putative targets. These results suggested that some miRNA detected in the mitochondria like let-7 familly (let-7b, c, d, e, f, i) and mir-133a could be involved in a mitochondrial mRNA silencing regulation.

## Discussion

The present study demonstrated for the first time the localization of pre-miRNA (pre-mir-302a, pre-let-7b) and miRNA in human mitochondria isolated from muscular cells. The results of the *in situ* hybridization were associated to significant detection of a set of miRNA in the mitochondria as it has been found in rat and mouse liver mitochondria [18]–[19]. Between 6 and 27% of the human miRNA panel could be significantly detected in the human mitochondria. The experiment was conducted on skeletal muscular cell cultures in order to maximize the mitochondria enrichment using a very specific method of isolation. The LNA *in situ* hybridization in the mitochondria was an original method derived from other protocols described for the *in situ* detection of miRNA. The method have been successfully used to study the specific regional co-localization of different miRNA in several type of cells or tissues such as rat myogenic cells and mouse fetal heart, mouse, human and monkey cerebellum, and human bladder biopsies [20]–[21]. The LNA technology enabled a highly sensitive and specific detection of pre-miRNA and miRNA. As it circumvents the need for mitochondrial purification, this approach helped to clearly establish the localization of at least two pre-miRNA (prem-mir-302a and pre-let-7b) and one miRNA (mir-365) in human mitochondria. These co-localization results were confirmed by the miRNA RTqPCR analysis. The mature form of let-7b was significantly detected by RT-qPCR in the mitochondria RNA extract but poorly localized in the mitochondria by *in situ* hybridization observed in confocal microscopy. The co-localization was stronger in the nucleus. Although we cannot completely eliminate a very low expression in the cells analyzed.

The issue of the potential cytosolic contamination of the mitochondrial fraction was carefully addressed in the present study. First, we coupled *in situ* hybridization of LNA specifically targeting miRNA to the specific marker of active mitochondria (MitoTracker®). This marker applied before fixative treatment is only active on live mitochondria. Thus, the staining was a guaranty of membranes integrity and no cytosolic contamination. In addition the use of both conventional optic and confocal microscopy to avoid the risk of co-localization error of the fluorescent markers in the mitochondria. Second, for the mtRNA extraction, we used a very selective method (MACS) of mitochondria isolation using outer membrane protein antibody anti-TOM22 conjugated to supramagnetic microbeads. Third, we applied both detergent and RNAse washes of the mitochondrial fraction.

The mitochondrial fraction was isolated with a very selective extraction method consisting of superparamagnetic microbeads conjugated to an outer membrane protein antibody against TOM-22. This innovative method was validated by comparison with two other methods [22]: differential centrifugation which is the most frequently used and ultracentrifugation on percoll gradient which is more sophisticated and expensive. It was found that the magnetic cell sorting (MACS) method provides better quality, purity and quantity of mitochondria fraction than the traditional differential centrifugation method. The performance of the MACS method was quite similar to the ultracentrifugation method with Percoll gradient. In particular, fewer contaminants of endoplasmic reticulum and nucleus were detected by antibodies in the mitochondria fraction compared to the differential centrifugation method. Western blot analysis revealed that the enrichment of isolated mitochondria by using TOM22 as marker was 59% in differential centrifugation, 88% in ultracentrifugation and 89% in MACS method. Another paper validated MACS mitochondria isolation method by western blot of the glycogen synthase, used as cytosolic marker, was completely absent in purified mitochondria while prohibitin, used as mitochondrial marker, was very enriched in the purified mitochondria [23]. After mitochondria isolation, we applied successive detergents and RNAse A washes to further reduced any residual cytosolic RNA contamination. The same approach was also applied in the two recent studies reporting on the presence of miRNA in mouse mitochondrial fraction [18]–[19]. In the present study, the enrichment of mtRNA in the mitochondrial fraction was carefully evaluated by four independent methods at the protein, mRNA, DNA and ultrastructure level. The very low cytosolic mRNA contamination and high mtRNA enrichment was confirmed at the protein level. We showed by western blotting analysis that two specific proteins from cytosol or cytoskeleton were poorly detected in our mitochondrial fraction whereas two specific mitochondrial proteins were highly enriched. Furthermore, the high mitochondrial to nuclear DNA ratio confirmed the high mitochondria enrichment. Finally, transmission electron microscopy revealed the membranes and ultrastructure integrity of many isolated mitochondria. All these evalutation results indicated that the mitochondria isolation protocol used in our study provided a high mitochondria enrichment fraction with fewer contaminants from cytosol or nucleus.

Although we clearly demonstrated the co-localization of at least two pre-miRNA and one miRNA, associated to the detection of a set of miRNA in the human mitochondria, we cannot conclude on the origin of these miRNA. Whether mitochondrial miRNA are transported into the mitochondria or endogenously synthesized remain unknown and should be further evaluated. Several arguments could support the miRNA importation hypothesis. Among the miRNA observed in the mitochondria, we detected a sub-group of “myo-miRNA” known to be involved in muscular cell proliferation and differentiation [24]–[26]. There was not any myo-miRNA sequence in the mtDNA and consequently, they had to be imported in the mitochondria by an unknown translocation system. Some of the myo-miRNA like mir-133a was predicted to have mitochondrial gene target sites according to our *in silico* results. ND1 has a target site for mir-133a that was detected in the mitochondrial fraction according to our RT-qPCR results. This miRNA is known for its muscular activity during differentiation of myoblasts in myocytes which was exactly the case of the cell culture in the present experiment [24]. Three other miRNA involved in muscles physiology, mir-1, mir-181a, mir-181b and mir-206 were detected in the mitochondria but we did not find any target site in mtDNA. The miRNA identified in the rat liver mitochondria were probably also imported and only one miRNA target was predicted on mitochondrial gene COX3 for the mir-130 [18]. Processes of RNA importation into the mitochnodria has been demonstrated in many organisms, including protozoans, plants, fungi, yeasts and animals. It has been used to treat MERF mitochondrial mutation in human fibroblasts by importation of cytosolic tRNA^Lys^ [27]. Unknown protein factor(s) could promote the importation of the 5S rRNA in human mitochondria [6]. It was also demonstrated that the cytosolic tRNA_Gln_ could be imported into mitochondria using ATP but does not require any cytosolic factor or the protein import system [7]. Another mechanism of tRNA importation in the mitochondria has been described in plants by voltage dependent anion channel VDAC which is located in the outer membrane and can translocate tRNA or proteins [28]. The importation of tRNA in the mitochondria is a natural process that could also play a role for the mitochondrial miRNA importation [29], [30]. Conversely, tansportation of a 70 nt RNA (tRNA^Met^) by argonaute Ago2 out of the mitochondria to the cytosol has been described in human [31]. The argonaute Ago2 is the main active protein of the RNA induced silencing complex (RISC) which has been recently isolated in the mouse mitochondria [19]. This finding suggested that Ago2 could be involved in the final step of the mitochondrial miRNA interference process.

The hypothesis of mitochondrial miRNA synthesis could be supported by the present results of co-localization of pre-mir-302a and pre-let-7b in the mitochondria. It was the first observation of pre-miRNA in the mitochondria. In addition, the bio-informatic results revealed many potential pre-miRNA sequences including pre-mir-302a and pre-let-7b in the mitochondrial genome. This result may suggests that some pre-miRNA sequences seemed to be processed in the mitochondria may be to synthesize mature miRNA which could be immediately active on the mitochondrial transcripts or exported in the cytosol in order to interfere with genomic mRNA. Thus, the mitochondrial miRNA may contribute to some post-transcriptional regulation of gene expression related to the main mitochondrial functions (energetics, apoptosis).

Finally, it was interesting to annotate some of the miRNA observed in the mitochondria. Even if it was not the main objective of the study, RT-qPCR on highly purified mitochondria extract allowed to make a first list of at least 46 mitochondrial miRNA detected in muscular cells. We identified five main miRNA functions: myogenesis, inflammation, fibrosis, oncogenic and onco-suppressor activities. The mir-133a is involved in the proliferation regulation of the myoblasts and then the differentiation in myotubes is regulated by the mir-1, mir-206, mir-181a and mir-206 [24]–[26]. The mir-29a was up-regulated in hepatic or systemic fibrosis [32], [33]. The mir-365 up-regulation was associated to the inflammation pathway [34], [35]. The mir-31 [36]–[38], mir-302a [39], [40], mir-21 [41], mir-181b [41], [42] and were found to be up-regulated in several cancers. By contrast, let-7b which belongs to the let-7 family was known as a tumor suppressor by reducing the expression of RAS oncogene [43]–[47]. Several other members of the let-7 familly were significantly detected in the mitochondria. The transcription factor p53 has been also co-localized in the mitochondria during p53-dependant apoptosis and is a putative regulator of let-7 familly and other miRNA (mir-107, mir-145, mir-134, mir-503, mir-21) detected in the mitochondria [48], [49]. In addition, p53 regulates mircroRNA processing machinery (Drosha, Dicer, DGCR8, TARBP2, Exportin5) [49]. Thus, the miRNA localization in the mitochondria, like let-7b and other let-7 miRNA, could be related to the apoptosis regulation as it was suggested previously [18], [19]. The mitochondria were suspected to be involved in tumorogenesis [50] and unbalanced transcription activity was discribed in breast cancer where COX2 is up-regulated compared to non-malignant tissue while ND2, ND4 and ATPase6 are unchanged [51]. The post-transcription regulation by miRNA interference could be one explanation of the unbalanced number of mitochondrial gene transcripts which are processed all together in a one long transcript and then cleaved in several independent gene transcripts.

The present study experimentally demonstrated for the first time the presence of pre-miRNA and mature miRNA in the human mitochondria of skeletal muscular cells. The *in situ* hybridization clearly localized two pre-microRNA (pre-mir302a and pre-let-7b) and one microRNA (mir-365) in the mitochondria. This co-localization results were confirmed by the detection of at least 46 miRNA significantly detected in the purified mitochondria RNA extract. Bio-informatic analysis of the mtDNA reference sequence allowed to detect putative miRNA target sites which could partly explain the presence of miRNA in mitochondria. The origin of these pre-miRNA and miRNA should be further investigate to determine if they are imported from the cytosol using an unknown translocation system, and/or they could be processed in the mitochondria as it could be suggested by the findings of the two pre-miRNA (pre-let-7b and pre-mir-302a). Finally, post-transcription regulation by RNA silencing in the mitochondria may exist if the miRNA enzymatic machinery could be imported and active in this organelle which should be further demonstrated. Our results open a new avenues of research regarding the miRNA functions in the mitochondria and cross-talk with nucleus in normal and pathological cells.

## Materials and Methods

### Human primary myoblast culture and *in-vitro* differentiation in myotubes

The cryopreserved human skeletal muscle myoblasts (HSMM, Lonza) were placed in tissue culture flasks at a seeding density of 3500 cells/cm^2^. Growth medium contained fetal calf serum and was supplemented with growth factors: rhEGF, 0.5 mL; Dexamethasone, 0.5 mL; L-Glutamine, 10 mL; FBS, 50 mL; GA-1000, 0.5 mL (SkBM-2 Basal Medium and a SingleQuot®, Lonza). Culture was maintained in an incubator equilibrated with 5% CO_2_ at 37°C. When the skeletal myoblast culture reached 50–70% confluence, the cells were passaged to the density of 10 000 cells/cm^2^ in the same medium. A part of the myoblast culture flasks were used to perform in situ hybridization. At 50–70% confluence, myotube differentiation was induced by changing the medium and growing cells in DMEM supplemented with 2% horse serum for 3 to 5 days or until myotubes were observed. The medium was replaced everyday with fresh medium. Myotubes were collected by trypsinization and centrifuging at 1000 g for 10 minutes and collected in 1 mL of RNAlater ® (Ambion). Myotubes were then frozen until mitochondria isolation at −80°C. The myotubes culture flasks were used for mitochondrial RNA extraction, the small RNA quantification and the miRNA detection by RT-qPCR.

### *In situ* hybridization probes

Locked nucleic acid (LNA) hybridization probes complementary to human mature miR-let7b (5′-ACCACACAACCTACTACCTC-3′), miR-365 (5′-AGGATTTTTAGGGGCATT-3′), and to precursor of miR let7b (5′-TATCTTCCGAGGGGCAACA-3′), precursor of miR 302a (5′-GAAGCACTTACTTCTTTAGTTTC-3′) were provided from Exiqon (Vedbaek, Denmark). A negative non-hybridizing control named scramble miRNA (5′-GTGTAACACGTCTATACG CCCA-3′) and a LNA U6 small nuclear RNA positive control probe (5′CACGAATTTGCGTGTCATCCTT-3) have been also purchased from Exiqon. Two type of probes were provided: probes both 5′ and 3′ digoxigenin-labelled and probes without digoxigenin-staining.

### Hybridization *in situ* and immunofluorescence microscopy

HSMM cells (Human Skeletal Muscle Myoblasts from Lonza CAT : CC-2580) were plated on 18 mm coverslips in 12-weel tissue culture dishes in SkGM®-2 medium containing 0.1% rhEGF, 0.1% Dexamethasone, 2% L-glutamine, 10% FBS, 50 ml; 0.1% GA-1000 (SkGM®-2 BulletKit® ; CC-3245). Cells were cultured at 37% in 5% CO2 reaching a confluency of 60–70%. Then, 3 µM MitoTracker® Red CM-H_2_XRos (Invitrogen, ref M-7513, Molecular Probes, Leiden, The Netherlands) treatment was conducted on live HSMM cells during 45 min at 37°C, 5% CO2 to visualize respiring mitochondria. The coverslips were rinsed twice with DPBS (5 mM MgCl2). Cells were fixed in 4% paraformaldehyde/5 mM MgCl2 in DPBS. In situ hybridization with 2.5 pmol of LNA probes and washed were performed as previously described [21]. Then, the coverslips were then processed for indirect immunofluorescence staining using a secondary antibody anti-digoxigenin conjugated with fluorescein (Roche, Cat. No. 11 207 741 910). Finally, nuclei were stained using Hoechst 33342. The coverslip were mounted on glass slides with Dako antifading mounting solution (Invitrogen).

### Immunofluorescence and confocal microscopy

Images were obtained on the hand with an Olympus BX61 straight microscope controlled by Metamorph software (Molecular Devices, Downington, PA19335) using a 100× oil-immersion objective. On the other hand images were obtained with a Leica TSC-P2 confocal microscope using a 63× oil-immersion objective and a sequential mode for three colour acquisitions (FITC, dapi and Cy3) were used. The images were analyzed using Image J software. The percentage of pixels from functioning mitochondria co-localized with pixels from specific LNA probe signals for mir or pre-mir were determined using Isodata plugging threshold (Image J software) and indicated on the figure (lane 5).

### Isolation of mitochondria by magnetic antibody cell sorting method (MACS)

Mitochondria were isolated from human skeletal muscle cells using supramagnetic microbeads conjugated to anti-TOM-22 antibody according to the validated isolation method [23]. Briefly, cells were lysed, and mitochondria were magnetically labeled with anti-TOM22 antibody microbeads. This monoclonal antibody specifically binds the translocase of the outer mitochondrial membrane 22 (TOM22) of human mitochondria. The labeled cell lysate was loaded onto a column placed in a magnetic field separator. The magnetically labeled mitochondria were retained in the column during washing. Then the magnet is removed from the column support and mitochondria were eluted. Pure, functional mitochondria were collected, centrifuged at 13000 g for 2 minutes at 4°C and the supernatant was discarded. The pellet was resuspended in 100 µL RNAlater ® (Ambion) until miRNA RT-qPCR analysis. Some mitochondria pellets were resuspended in 100 µL of storage buffer (MACS, Miltenyi) for transmission electronic microscopy preparation and western blotting of mitochondrial proteins.

### Transmission electron microscopy to control the mitochondria integrity

The mitochondria pellets were fixed with 2% glutaraldehyde in 0.1 M Na cacodylate buffer pH 7.2, for 4 hours at room temperature and then postfixed with 1% osmium tetroxide containing 1.5% potassium cyanoferrate, contrasted with uranyl acetate 2% in water, gradually dehydrated in ethanol (30% to 100%) and embedded in Epon (Delta microscopie – Labège France). One micron sections were collected onto glass slides, counter stained with methylen blue-Azur II. Sections were imaged on an epifluorescent microscope (DMRB – Leica - France) with a 63× plan apochromat oil immersion lens. Acquisition was performed using a CCD camera (Olympus DP50) and processed with adobe photoshop CS software (VWR – France). Thin sections (70 nm) were collected onto 200 mesh cooper grids, and counter stained with lead citrate before examination with Zeiss EM902 electron microscope operated at 80 kV– (MIMA2- UR1196 Génomique et Physiologie de la Lactation, INRA, Plateau de Microscopie Electronique 78352 Jouy-en-Josas, France). Microphotographies were acquired with a charge-coupled device camera MegaView III CCD camera and analysed with ITEM software (Eloïse – SARL – Roissy CDG – France).

### Mitochondria fraction decontamination of cytosolic RNA

The mitochondria were washed with RNAase A solution in order to remove all the RNA of nuclear origin present in the cytosol which could be adsorbed on the outer mitochondrial membranes. The RNAse solution was a mixture of 4 µL of RNAse A (10 µg/µL) and 994 µL of a resuspension buffer P1 (first part of the Qiagen plasmid purification protocol). We added 300 µL of this RNAse A solution to 20 µL of resuspended mitochondria. The mix was incubated at 37°C for 1 hour. Then, the RNAase activity was stopped with another solution including 5 µL of proteinase K (20 mg/mL, Qiagen).

### Mitochondrial RNA extraction

The washed mitochondria were resuspended in 100 µL lysis solution and vortexed vigorously for mtRNA extraction according to a specific kit for total RNA extraction of small amounts of cells (RNAqueous ®Microkit, Ambion). Then, the total mtRNA was quantified with a spectrophotometer (Nanodrop 1000 ®, Thermo Scientific).

### Control of the mitochondrial enrichment and cytosolic contamination

Three tests were performed to assess the mitochondria enrichment and the risk of cytosolic contamination at the mitochondrial protein, mRNA and DNA levels. Protein levels were determined by western blotting analysis using anti β-actin-peroxidase (Sigma ref A3854), rabbit GAPDH (glyceraldehyde-3-phosphate dehydrogenase, Santa Cruz), mouse monoclonal anti-TOM22, (22-kDa translocase of outer mitochondrial membrane, Miltenyi Biotec) and rabbit polyclonal ATP synthase (generous gift of G. Brandolin) as primary antibodies. The western blot protocol and secondary antibodies used have already been extensively detailed and validated [52]. The signal generated by the different amounts of proteins were quantified by Gelpro32 Analyser.

One part of the mitochondria total RNA extract was used to check the quality and purity of the mitochondrial fraction. The primers of the following two mitochondrial genes (ND4 and CYTB) and two nuclear genes (HIST2H2AA3 and GAPDH) were used for both transcripts expression and DNA relative quantification:

MT-ND4:

fwd:ACAAGCTCCATCTGCCTACGA

rev:GGCTGATTGAAGAGTATGCAATGA

MT-CYB:

fwd: AACCGCCTTTTCATCAATCG

rev: AGCGGATGATTCAGCCATAATT

Histone HIST2H2AA3 (Ref: Hs00358508_s1, TaqMan®, Applied Biosystems)

GAPDH (Ref: Hs99999905_m1, TaqMan®, Applied Biosystems).

After reverse transcription, the PCR reaction was analyzed by real time quantitative PCR (RT-qPCR) using fast real time PCR system 7300 (Applied Biosystems). The cycle threshold (CT) were normalized using the GAPDH as endogenous gene.

One part of the mitochondria fraction was used to extract mtDNA in order to check the mitochondria enrichment and the genomic contamination. DNA amplicons of the two same mitochondrial genes were obtained using the same primers. DNA amplicons of the two same nuclear genes were obtained using the same commercial primers. The amplicon concentrations were measured by RT-qPCR (fast real time PCR system 7300, Applied Biosystems). GAPDH was used to normalized the CT data and HIST2H2AA3 was used as a calibrator to calculate the mitochondrial to nuclear DNA ratio.

### Analysis of the mitochondrial small RNA concentration by microfludic electrophoresis

Small mitochondrial RNA concentration was measured in 9 samples of the mitochondrial fraction and 12 samples of the human miRNA reference panel including 735 different human miRNA (*mir*Vana® miRNA Reference Panel v9.1, Ambion). It was a microfluidic lab-on-a-chip technology using electrophoresis and dye to separate and detect RNA of 0 to 150 nt and the percentage of miRNA which was shorter than 40 nt (Small RNA kit, 2100 Bioanalyzer®, Agilent Technologies) [53].

### MicroRNA detection in mtRNA extracted from isolated mitochondria

RT-qPCR analyses were performed by the high quality service of Exiqon® (Vedbaek, Denmark). Three increasing samples of the mitochondrial RNA extract (1, 5 or 35 µl total RNA) were reverse transcribed in 40 µl reactions and diluted 100 fold in the RT-qPCR analysis using the miRCURY LNA™ Universal RT microRNA PCR, Polyadenylation and cDNA synthesis kit (Exiqon). Each microRNA was assayed once by RT-qPCR on the microRNA Ready-to-Use PCR, Human panel I and panel II including 742 miRNA (miRBase 13); the primers' description is available at: http://www.exiqon.com/mirna-pcr-primer; http://87.63.99.75/shop/excel-downloader-for-qpcr-or-unirt.jsp?type=unirt). Negative controls excluding enzyme from the reverse transcription reaction was performed and profiled like the samples. The amplification was performed in a LightCycler® 480 Real-Time PCR System (Roche) in 384 well plates. The LightCycler® 480 software was used to determine the Cp value. Each PCR panel contained a PCR control (three replicates of an inter-plate calibrator) and 3 primer sets for reference genes.

An additional step in the RT-qPCR analysis was performed to evaluate the specificity of the assays by generating a melting curve for each reaction. The appearance of a single peak during melting curve analysis is an indication that a single specific product was amplified during the RT-qPCR process. The appearance of multiple melting curve peaks correspondingly provides an indication of multiple PCR amplification products and is evidence of a lack of specificity. Any assays that showed multiple peaks have been excluded from the data set. The melting temperature was furthermore checked to be within the specifications for the individual assays. The list of miRNA excluded the sample exhibited multiple peaks during melting curve analysis.

The amplification curves were analyzed using the LC software (Roche), both for determination of Cp (by 2nd derivative method) and for melting curve analysis. All assays were inspected for distinct melting curves and the Tm was checked to be within known specifications for the assay. Assays must have Cp <35 to be included in the data analysis. Data that did not pass these criteria were omitted from any further analysis.

### Bio-informatic research of miRNA sequences and target sites in the mitochondrial reference sequence

In order to investigate the potential pre-miRNA and miRNA sequences in the mitochondrial genome, we used the miRBase sequence algorithm and the miR database (6396 miRNA, release 11) [54]. Alignments were performed between the reference mitochondrial sequence (AC_000021.2 GI:115315570) cut into tiling sequences 70 nt long and the whole miRNA database using SEARCH algorithm adapted for short sequences. The cut-off E-value was set to 0.1 (http://microrna.sanger.ac.uk/sequences/search.shtml). In addition, we scanned miRNA targets in the mitochondrial reference sequence (AC_000021.2 GI:115315570) in order to know if the miRNA silencing machinery could be efficient on some mitochondrial genes which may support the hypothesis of miRNA importation and/or maturation in the mitochondria. We used the miRBase target algorithm with a cut-off E-value set to 0.1 (http://microrna.sanger.ac.uk/targets/v5/).

## Supporting Information

Figure S1 **Enrichment of mitochondria proteins using MACS methodology.** The levels of ATP synthase, TOM22, GAPDH and ACTB were determined in the whole cell lysate and in mitochondrial proteins isolated from human myoblasts using MACS kit method. Equal amounts of proteins were loaded. The signal generated by the different amounts of proteins was quantified by Gelpro32 Analyser and the ratio mitochondrial proteins/whole cell lysate are indicated on the right of the gel.
(PS) Click here for additional data file.

Figure S2 **Transmission electron microscopy of isolated and purified mitochondria.** The images confirmed the integrity of the inner, outer membranes and the cristae ultrastructure after isolation using MACS method and fixative treatment. (A) & (B) show two isolated mitochondria with normal cristae, matrix density. (C) shows illustrated the integrity of the outer and inner membranes during a fission of two small mitochondria.
(TIF)

Figure S3 **Microfluidic electrophoresis of the mitochondrial RNA extract.** The spectrum shows the small RNA profiles in mitochondrial fraction (A) in comparison with the miRNA reference panel (B). (A) Microfluidic electrophoresis revealed several peaks of small RNA: one peak at 40–45 nt and another peak at 60–65 nt. According to the surface under the curve below 40 nt, 61% of miRNA were detected in this sample. (B) In comparison with the miRNA reference panel, we observed a small and smooth peak at 40 nt corresponding to the miRNA which was a sharp and high peak in the control miRNA panel presented in B.
(TIF)

Figure S4 **MiRNA detection in the mitochondrial mRNA extract in three increasing mRNA inputs.** The linearity of the assays was generally very good. The Cp (2^nd^ derivative) of each RT-qPCR reaction is shown as a function of the highest input RT-qPCR. As expected there is a linear parallel shift towards higher Cp as the input is decreased. As expected the variation increases with decreasing input of RNA. These results confirmed the quality of the mtRNA extraction for miRNA profiling.
(PS) Click here for additional data file.

Figure S5 **Quality control of microRNA RT-qPCR.** The positive controls were highly expressed in the three increasing mRNA inputs.
(PS) Click here for additional data file.

Table S1 **Quantification of the mitochondrial to nuclear DNA ratio in the mitochondrial fraction using two mitochondrial (ND4, CYTB) and two nuclear genes (HIST2HAA3, GAPDH).** GAPDH was used to normalize the results and HIST2HAA3 as calibrator to calculate de DNA ratio. This result shows the high enrichment in mitochondria and absence of nuclear DNA contamination of the mitochondrial fraction.
(XLS)

Table S2 **Relative expression of two mitochondrial genes (ND4 and CYTB) and two nuclear genes (histone HIST2HAA3 and GAPDH) in the mitochondrial RNA extract relative to the cytosolic RNA extract.** This result showed the low contamination level of the mitochondrial fraction by genomic mRNA.
(XLS)

Table S3 **List of microRNA significantly detected (Cp<35) in the mitochondrial mRNA at the two highest mtRNA inputs (5 and 35 µL).** Assays must have Cp<35 and distinct melting curves to be included in the list. Any assays that showed multiple peaks have been excluded from the data set. Fifty four and 160 miRNA were significantly detected for the two lowest mRNA inputs, respectively. It represented 27% of the human miRNA set tested.
(XLS)

Table S4 **Potential mitochondrial gene targets of human miRNA predicted bio-informatic algorithm of miRBase target.** The corresponding miRNA significantly detected in the mitochondria are indicated in bold and a mark in the column ‘tested miRNA’.
(XLS)
