# Supplementary figures and images for: Pre-microRNA and Mature microRNA in Human Mitochondria
Source: PLoS One. 2011 May 26;6(5):e20220. doi: 10.1371/journal.pone.0020220 (PMC3102686; doi:10.1371/journal.pone.0020220)

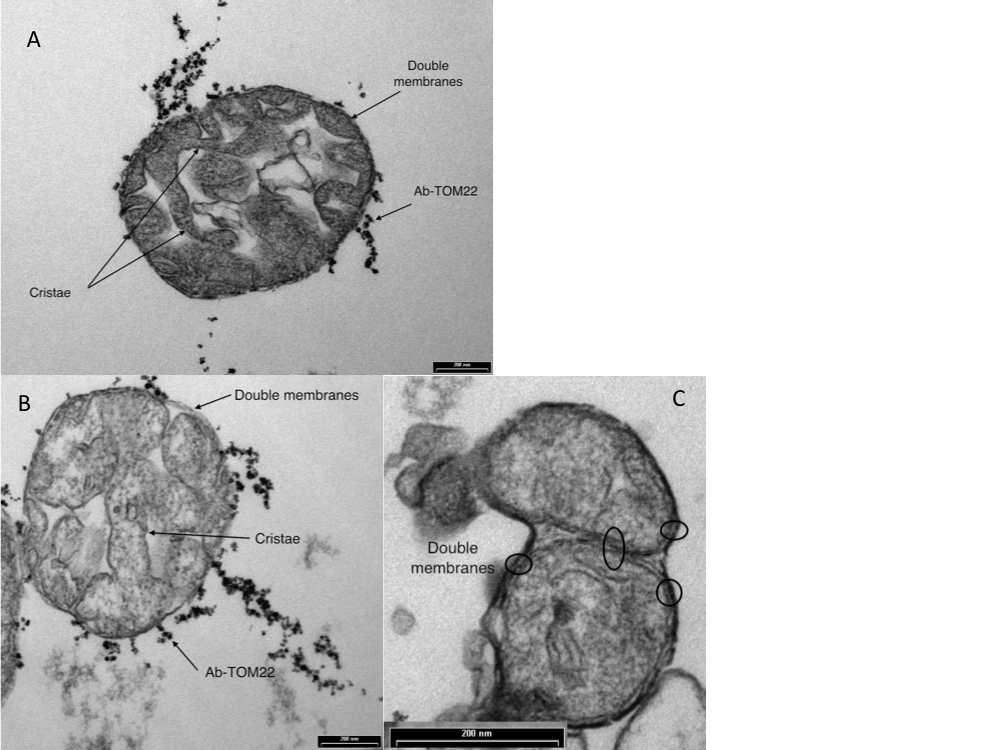

Supplement: Figure S2 — Transmission electron microscopy of isolated and purified mitochondria. The images confirmed the integrity of the inner, outer membranes and the cristae ultrastructure after isolation using MACS method and fixative treatment. (A) & (B) show two isolated mitochondria with normal cristae, matrix density. (C) shows illustrated the integrity of the outer and inner membranes during a fission of two small mitochondria. (TIF) [file pone.0020220.s002.tif]

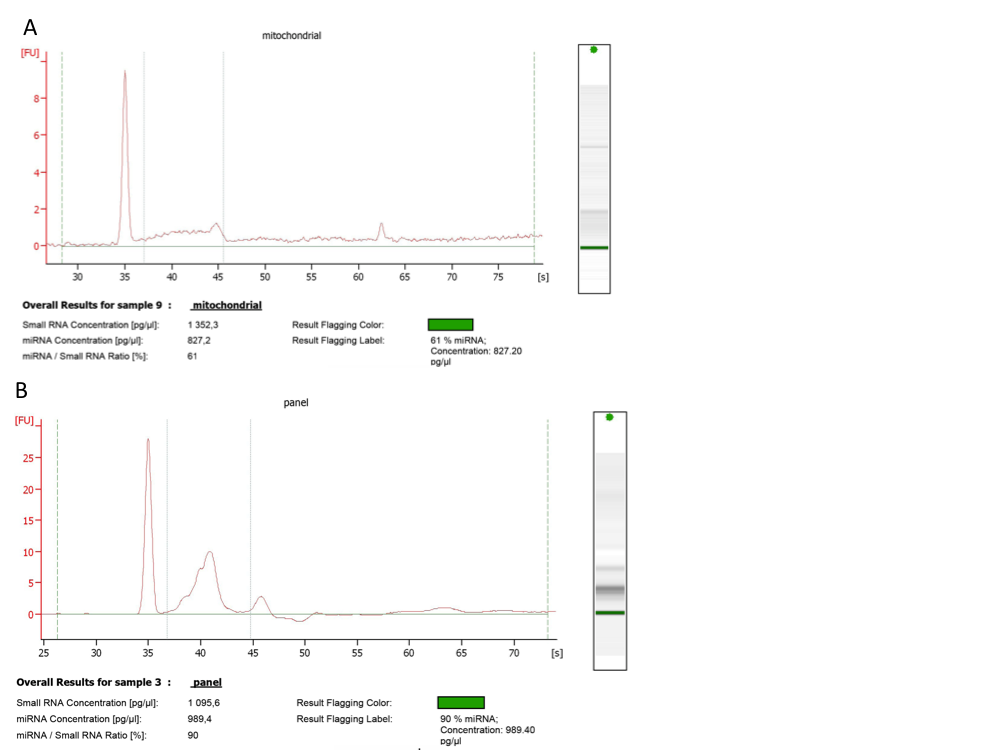

Supplement: Figure S3 — Microfluidic electrophoresis of the mitochondrial RNA extract. The spectrum shows the small RNA profiles in mitochondrial fraction (A) in comparison with the miRNA reference panel (B). (A) Microfluidic electrophoresis revealed several peaks of small RNA: one peak at 40–45 nt and another peak at 60–65 nt. According to the surface under the curve below 40 nt, 61% of miRNA were detected in this sample. (B) In comparison with the miRNA reference panel, we observed a small and smooth peak at 40 nt corresponding to the miRNA which was a sharp and high peak in the control miRNA panel presented in B. (TIF) [file pone.0020220.s003.tif]
